# T cell receptor sequence clustering and antigen specificity

**DOI:** 10.1016/j.csbj.2020.06.041

**Published:** 2020-08-05

**Authors:** Milena Vujovic, Kristine Fredlund Degn, Frederikke Isa Marin, Anna-Lisa Schaap-Johansen, Benny Chain, Thomas Lars Andresen, Joseph Kaplinsky, Paolo Marcatili

**Affiliations:** a DTU HealthTech, Department of Health Technology, Technical University of Denmark, Ørsteds Plads, Building 345C, DK-2800 Kgs. Lyngby, Denmark; b UCL Division of Infection and Immunity, University College London, Wing 3.2, Cruciform Building, Gower Street, London WC1E 6BT, United Kingdom; c Ludwig Institute for Cancer Research Ltd, University of Oxford, Nuffield Department of Medicine, Old Road Campus Research Building, Roosevelt Drive, Oxford OX3 7DQ, United Kingdom

**Keywords:** T cell receptor (TCR), Clustering, Epitope specificity, T cell receptor distance, T cell receptor similarity, T cell repertoire

## Abstract

There has been increasing interest in the role of T cells and their involvement in cancer, autoimmune and infectious diseases. However, the nature of T cell receptor (TCR) epitope recognition at a repertoire level is not yet fully understood. Due to technological advances a plethora of TCR sequences from a variety of disease and treatment settings has become readily available. Current efforts in TCR specificity analysis focus on identifying characteristics in immune repertoires which can explain or predict disease outcome or progression, or can be used to monitor the efficacy of disease therapy. In this context, clustering of TCRs by sequence to reflect biological similarity, and especially to reflect antigen specificity have become of paramount importance. We review the main TCR sequence clustering methods and the different similarity measures they use, and discuss their performance and possible improvement. We aim to provide guidance for non-specialists who wish to use TCR repertoire sequencing for disease tracking, patient stratification or therapy prediction, and to provide a starting point for those aiming to develop novel techniques for TCR annotation through clustering.

## 1 Introduction

Understanding T cell biology has long been essential to the study of infectious and autoimmune diseases. More recently, as immunotherapy has joined the traditional pillars of surgery, chemotherapy and radiation, it has also become more and more central to cancer biology.

The advent of high throughput sequencing has opened a new window on to the T cell receptor (TCR) repertoire. While there is much scope for improvement in TCR repertoire sequencing, these experiments are becoming increasingly routine. Two technological developments can be highlighted. First, the commercial availability of repertoire sequencing as a service and in the form of kits. Second, the availability of single cell sequencing. This allows the linking of the *α* and *β* (or *γ* and *δ*) chains of the TCR, while linking this TCR sequence to a phenotype such as memory or regulatory cell through single cell RNA-seq. Finally, the development of unique molecular identifiers allows for quantitation from sequencing data unbiased by PCR amplifications steps [1]. Because these technologies are now well established, T cells have been sequenced in a plethora of therapeutic and disease settings, as well as healthy control groups, and the data has been deposited on online databases such as the Sequence Read Archive (SRA) [2], VDJdb [3], TCR3d [4] and ImmuneACCESS database [5]. Most sequencing data available are still bulk unpaired *α* and *β* TCR sequences, due to the lower throughput and much higher cost of single-cell sequencing platforms.

However, the outstanding question in TCR repertoire analysis remains understanding the relationship between TCR sequence and TCR binding specificity. Sequence data itself contains no direct information on epitope specificity involved. While this may contribute towards models of sequence-binding specificity it will require more focused data sets to make substantial progress. *In silico* annotation of TCR specificity, would, for example, allow tracking of the number and expansion of clones that respond during the natural history of a disease, after vaccination, or during therapy. An example of this application would be to track ‘epitope spreading’ in response to cancer immunotherapy [6], [7].

**Fig. 1:**
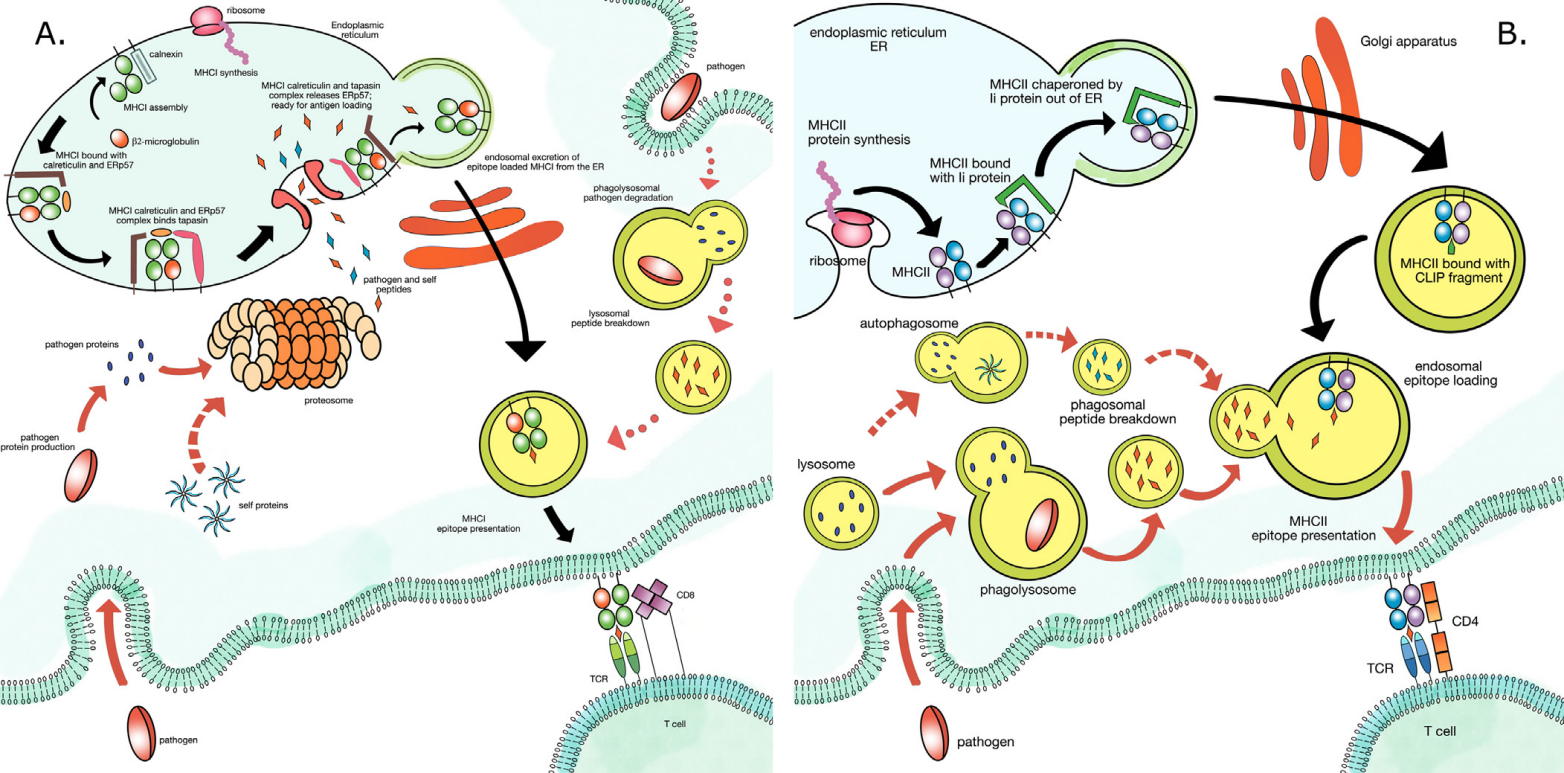
Schematic representation of MHC antigen processing and presentation adapted from cellular and immunobiological textbooks by Janeway [8] and Abbas et al. [27]. The MHC class I or II antigen presenting molecule comes into contact with CD8^+^ or CD4^+^ T cells, respectively. The binding to the T cell receptor (TCR), which induces T cell activation, is aided by the CD8 or CD4 protein, for MHC class I or II binding mechanisms, respectively. In both figures black arrows follow MHC synthesis and antigen presentation pathways. Red arrows follow antigen processing: solid - foreign-antigen direct presentation pathway; dashed - self-antigen direct presentation pathway; dotted - foreign antigen cross-presentation. **A. MHC class I antigen processing and presentation.** MHCI synthesis is started off by the ribosomes in the endoplasmic reticulum (ER). Additional incorporation of *β*2-microglobulin into the MHCI structure is aided by a transitional complex with the auxiliary protein calnexin. To protect from unsolicited interactions, the newly synthesised MHCI is complexed with calreticulin and ERp57, and subsequently to tapasin which will assist in epitope binding. Upon transporter associated with antigen processing (TAP) protein activation antigens come through into the ER and simultaneously the MHCI-tapasin-calreticulin complex releases ERp57 and widens the peptide binding cleft which allows for binding of compatible epitopes. The loaded complex is released from ER by endosome encapsulation and transported to the cell membrane to be expressed on cell surface. **Foreign and self antigen processing**. Some pathogens survive internalisation and continue to produce proteins in the cytosol. Alternatively, pathogens may be internalised along with their protein product. These proteins are degraded by the proteosome into peptide fragments, epitopes, and sent to the ER for peptide-MHCI assembly and presentation. Foreign epitopes are shown in orange. Self proteins follow a similar pathway of proteosomal degradation and are sent to the ER for peptide-MHC assembly and self presentation. Self epitopes are shown in blue. All nucleated cells express MHCI and follow these pathways for endogenous antigen presentation. **Cross-presentation**. Exogenous antigens are usually presented on MHCII expressing cells. In order to allow for MHCI presentation of exogenous antigens specialised cells process pathogens as in the MHCII pathway, but present on MHCI complexes. Several pathways might be involved in this process. The pathogen is first internalised and enzymatically degraded in the phagolysosome. The lysosome containing peptide antigens then comes into contact with synthesised MHCI molecule and form the peptide-MHCI complex. One possible pathway is that the generated antigens are transported from the lysosome, through TAP and are loaded onto the MHCI in the ER, following which they are expressed on the cell surface. Another pathway might include a vesicular loading compartment detaching from the ER, carrying the synthesised MHCI molecule, and merging with the epitope carrying lysososme. Upon merging the epitopes could load onto the MHCI and express onto the cell surface. **B. MHC class II antigen processing and presentation.** Pathogens are phagocytosed into the cell interior. Upon merging with a lysosome, proteases cleave the pathogen into short peptide fragments - foreign epitopes, here shown in red. The same fate befalls the cells own proteins as they undergo degradation by the autophagosome, leaving a phagosome containing short peptides - self epitopes, here shown in blue. Meanwhile, the MHCII protein is synthesised by ribosomes in the ER. Upon assembly, MHCII binds invariant chain, Ii protein. It prevents any unwanted protein binding to the MHCII complex in the ER. The Ii chaperones MHCII out of ER in an endosome. In the endosome, due to slightly acidic conditions the Ii protein degrades leaving class II associated invariant chain peptide, CLIP fragment bound in the MHCII cleft. Upon merging with a epitope containing phagosome, the MHCII comes into contact with foreign and self antigen fragments. Upon binding the peptide-MHCII complex is expressed on the cell surface where it is able to bind CD4^+^ T cells. (For interpretation of the references to colour in this figure legend, the reader is referred to the web version of this article.)

Antigens are presented to T cells in the form of short peptides via the major histocompatibility complex (MHC). There are two classes of MHC, class I, recognised by CD8^+^ T cells, and class II, recognised by CD4^+^ T cells. Antigen presentation via MHC I and MHC II differs, as shown in Fig. 1. While there is high overall homology between MHC I and MHC II, differences in structure and antigen processing results in shorter peptides (typically 8–13 amino acids) with buried ends presented on MHC I, than on MHC II (usually 10–22 amino acids) [8], [9], [10].

**Fig. 2:**
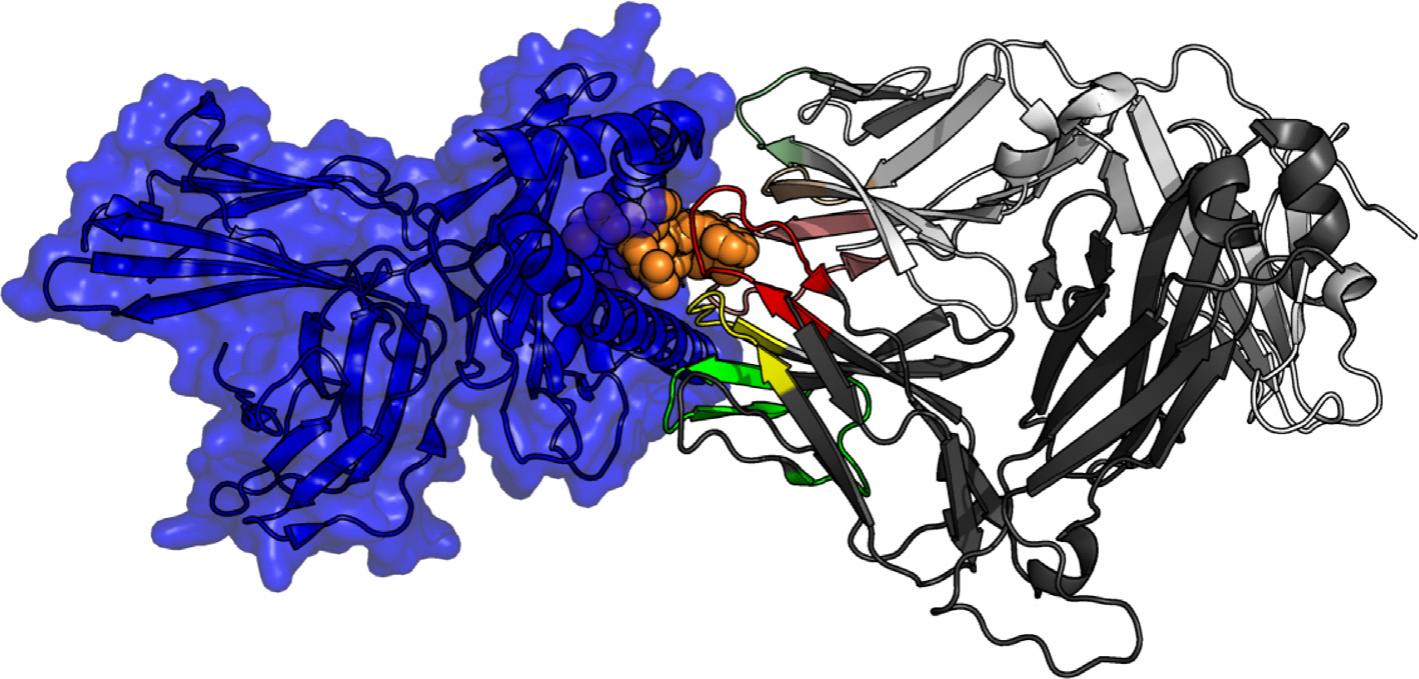
3D model of TCR-pMHC complex rendered via PyMOL [28],PDB reference code: 2BNR. MHC (blue) presenting peptide epitope (orange) comes into contact with the TCR (*α* chain light gray, *β* chain dark gray). Complementarity determining regions CDR1 (yellow), CDR2 (green) and CDR3 (red) come into contact with the pMHC. CDR3 comes into most contact with the presented peptide, while CDR1 and CDR2 on both chains mostly interact with the MHC. (For interpretation of the references to colour in this figure legend, the reader is referred to the web version of this article.)

To achieve high specificity and diversity of TCRs that allow for a directed response against a vast number of epitopes. TCRs undergo a stochastic process of V(D) J recombination in the thymus, through which they form three complementarity determining regions (CDRs) on each of the *α* and *β* chains [8]. TCR-pMHC complexes adopt diverse conformations, but in the majority of cases it is the loops formed by the CDRs which come into most direct contact with the peptide-MHC complex (pMHC), as shown in Fig. 2. In particular the CDR_*β*_3 loop, which is also the most diverse in sequence in the TCR, usually accounts for the largest part of contacts with the epitope.

The process of V(D) J recombination has the potential to generate an indefinite number of distinct TCRs. It is estimated that up to 10^20^ distinct TCRs can be generated with biologically significant probability [11], [12]. The human body contains on the order of 10^11^ T cells [13], and little overlap is generally observed between the repertoires in different individuals. It is therefore likely that each individual will respond with a unique set of TCRs to each epitope. A second important consequence of this extraordinary amount of sequence diversity is that many different sequences must code for TCRs which recognise the same epitope. Otherwise, many individuals would end up with no TCR for many antigens. In fact experimental measurements suggest that hundreds, or thousands of TCRs in each individual react with each peptide MHC complex [3].

On the other hand, there are several orders of magnitude more possible epitopes than T cells in an adult human [14]. Consequently, to provide protection against a broad spectrum of pathogens, the limited number of T cells within an individual must react with broad specificity towards foreign antigens, ignoring self, but simultaneously exhibiting cross-reactivity. In other words, many different TCRs must recognise the same peptide, but each TCR must recognise many peptides. This biological balancing act has made it difficult to understand which TCRs are responsible for an antigen response.

The most direct and detailed method for studying TCR-pMHC binding is X-ray crystallography. The progress in the field has provided very precise knowledge of some TCR-pMHC binding sites. The number of TCR-pMHC structures which have been solved is still limited (less than 100 unique currently available) [15], [16]. One approach to extend this data set is to use structural predictions, based on sequence. Despite the difficulties of modelling flexible loops, such as the CDR regions of the TCR, several tools have been explored, and the field is an active area of research. Models predicting TCR-pMHC binding based on their structure have already been investigated [15], [17], [18], [19], [20].

A number of other techniques probe the nature and quality of the T cell receptor interaction with pMHC. The ELISPOT assay [21] is one of the simplest methods for such an analysis, and has been widely used in assessing the quality of T cell responses. The surface of wells in a well-plate is coated with antibodies designed to capture cytokines secreted upon T cell activation. T cells are added to each of the wells, and upon addition of the antigen the number of activated T cells in each well and the magnitude of their response can be measured by the amount of bound cytokines surrounding each cell [22]. This analysis provides information on both the clonal size and the effector function of activated T cells. Despite the simplicity of the method, its major drawback is that no information is obtained on the TCR sequences of the T cells involved. Furthermore, the number of antigens tested in a single experiment is limited.

The key invention for sequencing of antigen-specific TCR subsets is labelled multimer technologies [23], [24], [25]. These allow for *in vitro* specificity testing and sorting of antigen specific T cells by binding to synthetic conjugates of peptide MHC (pMHC) molecules. The same restriction applies as with ELISPOT, in that there is a limited number of peptides that can be tested in this manner. However, unlike ELISPOT these T cells can be separated subsequently and sequenced to reveal information on nature of TCRs involved in a response to a single epitope. As the method is fully compatible with sequencing, it provides an unprecedented view into TCR-antigen specificity, by allowing simultaneous collection of information about both the epitope and TCR.

These experimental techniques provide abundant complementary data on TCR-epitope binding. Ideally, to make sense of this plenitude of sequence data one would like to be able to read out which epitope specificities are present in a sample, or in a more restricted way to test for reaction against specific epitopes, using sequence data alone. However, inferring this from primary sequence information is a challenging task as it involves prediction of protein-protein binding without knowledge of exact structures of proteins involved. Still, both TCRs and pMHCs have some defined structure with known variable regions and restricted number of binding conformations. As tertiary and quaternary structure of functional proteins is dependent on their primary sequence, it is reasonable to believe that protein-protein interactions could be inferred from the sequence information alone. Structure prediction of pMHC is relatively straight-forward, unlike the prediction of TCR structure which becomes quite the ordeal due to the high variability of the CDRs. Current TCR structure prediction tools such as LYRA [26] and TCRmodel [4] are able to predict TCR structure with a striking reported accuracy for a protein with such a high degree of variability, with benchmarked average RMSD accuracy of 1.48 Å reported for LYRA. Even though these predictions have not yet reached the accuracy of the related protein family of antibodies, the models are quite useful as they convey information about the true protein structure.

**Fig. 3:**
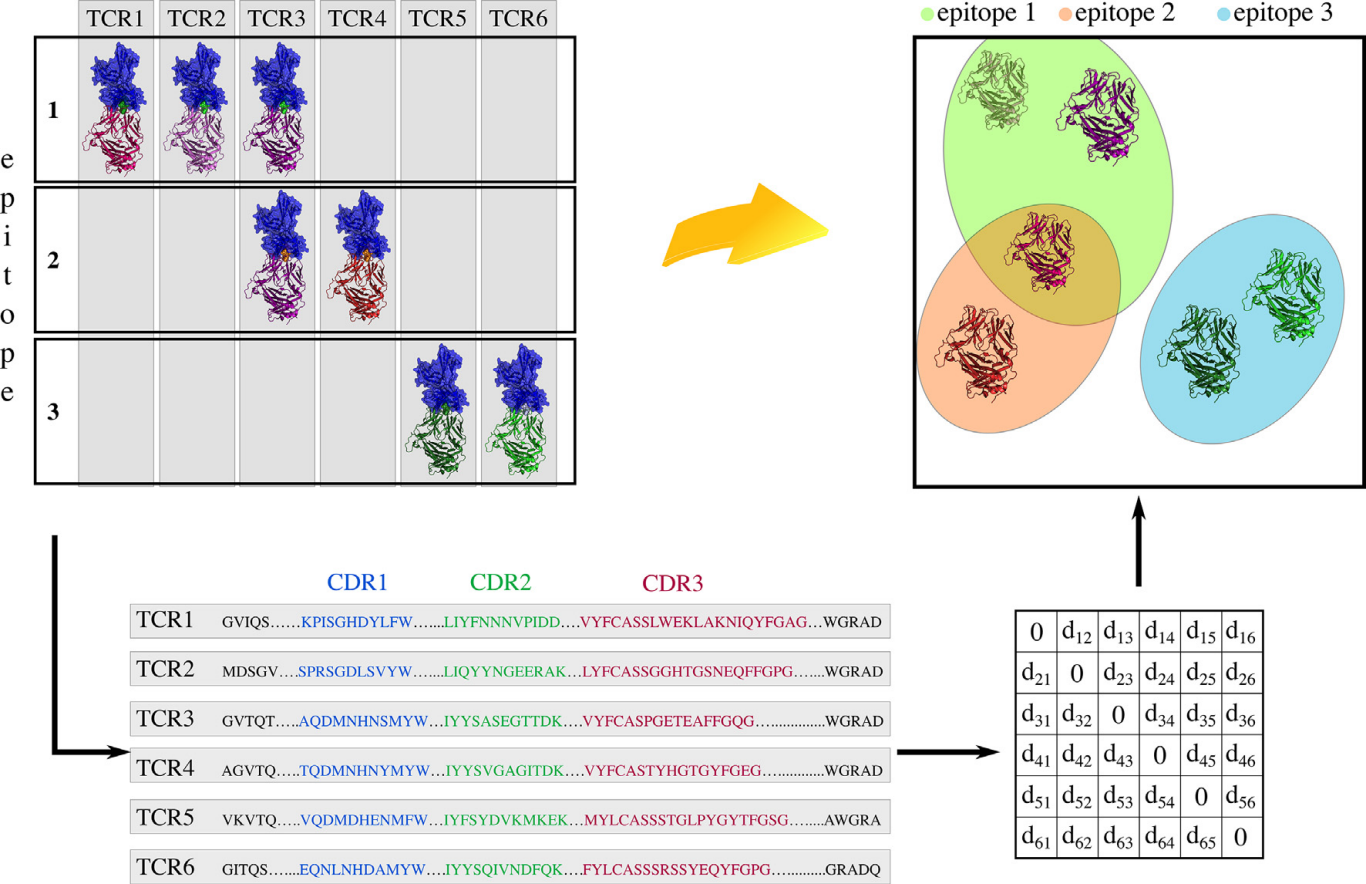
Graphical representation of attempts to encompass structural and sequence similarity in a suitable clustering distance metric that aims to capture epitope specificity. Binding of six fictional TCRs to three fictional epitopes is depicted on the upper left side. The TCRs are shown in shades of green, purple and red, while epitopes are coloured in green, orange and light purple. If primary sequences of the TCRs are known, sequence comparison can be used to create a distance matrix TCR distance matrix. The matrix could then be used to cluster individual TCRs together based on their sequence similarity, with the goal of clustering by biological similarity i.e. epitope response. (For interpretation of the references to colour in this figure legend, the reader is referred to the web version of this article.)

The main challenge is constructing a TCR comparison strategy that will somehow reflect the epitope specificites of TCRs involved, as illustrated in Fig. 3. Understanding the complex mechanisms of TCR antigen reactivity and expansion, could lead to correct patient stratification, track response to disease, help guide immunotherapy and further the development of precision medicine. Further, understanding the binding determinants might allow design of TCRs (or vaccines). Currently there are a number of approaches that aim to cluster TCRs by extrapolating information from their primary sequences to study their specificites.

In the remaining part of this review, we discuss the latest discoveries in the field of TCR specificity and repertoire analysis. We aim to provide a complete overview of all TCR clustering methods and repertoire analysis, their advantages and pitfalls,in hopes of facilitating the choice of data analysis choice for experimentalists and bioinformaticians alike. We aim to showcase all current TCR grouping strategies and their ability to translate into biological similarity or classification of repertoires. It is also our hope that outlining current state-of-the art will facilitate further development of improved TCR clustering techniques.

## 2 Sequencing based approaches

The largest experiments aimed at linking antigen to TCR sequence using multimer technology are now reaching trillions of TCRs [29]. The collaborative approach between Microsoft Healthcare NExT initiative and Adaptive Biotechnologies aims to provide a comprehensive mapping of T cell receptors and their antigen targets covering a multitude of diseases. They aim to unearth biologically and clinically relevant antigens across diseases that can be used for diagnostic purposes from a single blood test. The proof-of-concept study by Emerson et al. [30], outlined initial steps in an diagnostic classification of Cytomegalovirus (CMV) positive and negative individuals by TCR repertoire analysis. A Bayes probabilistic model, based on presence/absence of specific TCRs in 352 CMV negative and 289 CMV negative individuals was used to predict a binary classifier, CMV serostatus. The feature selection and model parameter selection was initially done using cross-validation to provide training and testing sets. The model was also tested on an external validation set of 120 subjects. The authors report excellent classification performance with an AUC of at least 0.93, based on a small (less than 200) set of TCRs over-represented in the CMV+ cohort. This study suggests the potential of TCR sequencing data in disease diagnostics, tracking and treatment in the future. However, it also suggests that very large sequence data sets will be required to provide sufficient power if presence/absence of specific sequences is used, without any attempt to cluster TCR sequences with similar epitope recognition.

### 2.1 Sequence alignment and clustering approaches

Algorithms which cluster TCRs (or often only CDR3 sequences) exploit similarity measures between TCRs with the aim to identify antigen specificity. In other words, members of a TCR sequence cluster should all recognise the same pMHC. Broadly, the approaches can be divided into those that use global similarities across the whole TCR or CDR3, and local similarities which focus on small amino acid motifs. A common approach of assessing global protein similarity is by sequence alignment and scoring using pre-calculated position specific scoring matrices, such as the BLOSUM [31] and PAM [32] family of matrices. There exist several alignment algorithms [33], [34], [35] which use a gap introducing penalty and a substitution matrix to align two sequences by their most similar or identical stretches. An important difficulty in alignment of TCRs with known specificities is that TCRs are cross-reactive and may bind multiple very different epitopes. Conversely, a single epitope may be bound by very different TCRs. Moreover, substitution matrices such as BLOSUM and PAM have been derived from studies of evolutionary related proteins. In this case, rather than serving as a measure for evolutionary relatedness of TCRs responding to the same epitope, such matrices provide a useful starting point as a proxy for physico-chemical similarity.

An example of sequence alignment approaches employed in TCR repertoire analysis is the ImmunoMap algorithm [36] (code available at: github.com/sidhomj/ImmunoMap). Sidhom et al. evaluate the CD8^+^ T cell response, from naïve and tumour bearing B6 mice, *in vitro* which bind either self tumor-associated antigen (Kb-TRP2) or a foreign tumor-associeted (Kb-SIY) antigen tetramer nanoparticle artificial antigen presenting cells (nanoAPCs). After *β* chain sequencing, they create a distance matrix between CDR3 regions using a PAM10 scoring matrix and a large gap penalty and further perform hierarchical clustering on the basis of this distance matrix. The novelty of their clustering approach comes from the visualisation of the dendogram, where the authors add intuitive endings to the branches corresponding to clone sizes. This approach revealed that in the naïve mice response to the self antigen, the expanded T cells in the repertoire were more unrelated and higher frequency than the T cells against the foreign antigen. In tumour bearing mice, the situation altered slightly in the self response with an observed elevated number of high frequency clones as well as usage of distantly related sequences. Following murine sequence analysis, the method was tested in 34 metastatic melanoma patients undergoing *α*-PD1 immunotherapy (Nivolumab), from whom Tumour Infiltrating Lymphocytes (TILs) were extracted and sequenced. Repertoires were compared prior- and post-therapy, and the authors report observing distinct features on the ImmunoMap dendogram between responders and non-responders, such as the number of high frequency clones and CDR3 relatedness. This was further corroborated by the dominant motif analysis from the expanded ImmunoMap detected clones, which showed some classification power. Although this analysis doesn’t seek to assign TCRs to particular epitopes, it conveys a notion of the importance of CDR3 similarity clustering. It also highlights the complexity of response towards even just a single epitope, as assessed by binding to multimer nanoAPC. This graphical approach proves very useful in displaying properties of repertoires with a single specificity; however, it fails to scale up and give an easily readable representation of repertoires at large.

A more focused effort in TCR clustering reflecting epitope specificity comes in the form of TCRdist by Dash et al. [37] (code available at: github.com/phbradley/tcr-dist). The authors used tetramer staining and single cell sequencing to obtain 4635 paired *α* and *β* TCR sequences from 10 different epitope specific repertoires. They analysed data from 78 mice and humans specific for murine and human cytomegalovirus (CMV), influenza and Epstein-Barr virus antigen epitopes. In order to analyse the data they constructed TCRdist, a distance metric based on both the *α* and *β* chain of the receptor. It is a similarity weighted mismatch distance using alignment with BLOSUM62 [31] substitution matrix to calculate similarity between CDR regions. Gap penalties are low for the CDR1 and 2 regions, but increase for the CDR3, stemming from the need to conserve short length motifs in the CDR3 regions which might be responsible for binding. Finally a distance between two TCRs is calculated by summing over scores for each CDR region on both chains, as well as an additional variable loop they term CDR2.5. The CDR3 loop scores on both chains is upweighted in the sum, since it is believed to contain most of the information about epitope binding. Using this TCR distance they proceed to cluster TCRs within each epitope-specific repertoire as well as assign TCR sequences from influenza-infected lungs without prior knowledge of their tetramer specificity using nearest-neighbour-distance classifiers. They managed to correctly assign 81% human and 78% murine sequences to their epitope specific repertoire. To the best of our knowledge this is the first specialised single cell TCR similarity measure which use combined *α* and *β* chains. However, one limitation of the clustering evaluation is that the metric has not been evaluated on complex repertoires originating from responses from multiple epitopes.

Another metric, CDRdist, developed by Thakkar et al. [38], takes solely CDR3 sequences into account (code available at: https://github.com/neerjathakkar/Distinguishing-TCR-Groups). The authors evaluate performance and separately apply their metric on CDR_*α*_3 and CDR_*β*_3 sequences. To evaluate sequence similarity CDRdist uses local alignment and a substantial gap penalty with BLOSUM45 [31] substitution matrix, usually used for more distantly related alignments than with the higher order BLOSUM matrices. Using this combination of parameters they allow for larger physico-chemical diversity, therefore generating longer matching substrings in the alignments. The authors proceeded to analyse data from monozygotic twins previously published by Zvyagin et al. [39]. The original analysis showed that the number of identical CDR3s shared between twins was significantly increased compared to non-twin individuals. Thakkar et al. broadened the hypothesis from considering identical sequences, to considering similar sequences, and in fact exclude identical CDR3 sequences from consideration. Applying CDRdist to each CDR3 in the repertoires, they evaluated whether the nearest CDR3 neighbour came from a twin, or another individual. As the number or nearest neighbours coming from twins outweighed those coming from other individuals, they reach the conclusion that twins have more shared similar sequences than non-twins. This finding is perhaps not unexpected, but it strengthened the belief that the CDRdist conveys biological meaning, before proceeding to the more difficult task of epitope classification. Following the approach of Dash et al. [37], they try to assign CDR3 sequences to their respective antigen specificity groups from the same epitope-specific repertoires used in Dash et al. by using the nearest neighbour distance classifier. The authors report comparable performance to TCRdist using only CDR_*β*_3 sequences, although they are not able to achieve the same result on CDR_*α*_3s. They achieve similar performance on the epitope-specific repertoires used for creating and evaluating the GLIPH algorithm [40] which is discussed at length further on. The authors also proceed to classify TCRs by which pathology they come from using data from McPAS-TCR catalogues [41]. They perform reasonably well on classification of infectious diseases (influenza, HIV, yellow fever and hepatitis C), but are not able to classify on cancers, autoimmune diseases and diabetes. Following closely the evaluation techniques of Dash et al. the authors do not evaluate their metrics classification power on a mixed epitope repertoire.

## 3 Analysis of characteristic short TCR motifs

The identification of short motifs within TCR sequences provides an alternative to the heavily parametrized sequence alignment and scoring approaches presented above. This approach is rooted in the hot spot interaction hypothesis, which states that only short stretches of complementary amino acid residues are responsible for epitope binding affinity [42], [43], [44]. Using short stretches of amino acids of length *k* (k-mers) in order to evaluate TCR receptor similarity could reduce informational noise, as opposed to comparing entire sequences. By focusing on short motifs, the problem of gaped alignment in TCRs of different lengths is also circumvented. By using k-mers in various forms, researches are able to pinpoint dominant motifs driving TCR-epitope specificity rather than individual expanded clones. One such approach is employed in the work of Thomas et al. [45] (code available as part of the Supplementary information of the same publication). In the study murine CD4^+^ T cells were bulk sequenced at different time points following immunisation with killed *Mycobacterium tuberculosis*. Every CDR_*β*_3 sequence was encoded as the list of all present triplets (k-mers of length 3). Instead of assessing triplet similarity using substitution matrices, the authors encode each triplet as a set of Atchley factors [46], corresponding to a set of physico-chemical properties. The authors then generate a *triplet codebook*, i.e. a reduced set of representative triplets to describe the complete pooled dataset. This is done by pooling and subsampling triplets from all samples, and grouping them by *k* means clustering. From each of the resulting clusters of similar triplets, a single representative triplet is selected in order to create the final triplet codebook. Each murine repertoire is then represented as a distribution of triplets in the codebook, by assigning each repertoire triplet vector to the most similar triplet in the codebook. Finally the repertoire representation is converted into a feature vector, used for classification using hierarchical clustering and Support Vector Machine (SVM) analysis [47]. Both techniques could classify immunised and non-immunised mice, but repertoires taken at different time points from immunised mice were not distinguishable. Although this study does not concern TCR-epitope classification, it highlights the importance of conserved characteristic motifs in assessing epitope responses. The authors note that their results reinforce imporance of diversity of the TCR repertoire, seeing as many private TCRs contribute to the T cell response to the same antigen in genetically identical mice. A subsequent study combined both global similarity metrics, and local amino acid motifs by Glanville et al. [40] This study evaluated publicly available CDR3s with known specificities, as well as their own pMHC tetramer sorted human CD4^+^ and CD8^+^ data (code available at: https://github.com/immunoengineer/gliph). They trained the GLIPH (Grouping of Lymphocyte Interactions by Paratope Hotspots) algorithm to search for enriched conserved TCR motifs of length 2, 3 and 4 within TCR multimer repertoires in the CDR_*β*_3 region. The distance metric then combines global and local TCR sequence similarity (CDR3s differing up to 1 amino acid and shared enrichment of motifs, respectively), V gene usage, CDR3 length bias, structural peptide antigen contact propensity and other features, with variable weightings for the different methods. GLIPH was evaluated on a mixture of 8 specificities, where it grouped 94% of the clustered TCRs together with others of same specificity. Another evaluation was performed on CD4^+^ *Mycobacterium Tuberculosis* specific T cells from 22 patients with latent *M. tuberculosis* infection. Clusters with TCRs shared between 3 individuals or more were examined, and found that 16 specificity groups that were shared between at least 3 individuals included at least 4 uniquely derived *β*TCR clones. This showed that enrichment of motifs can organise TCRs within or across individuals. Most importantly, the authors state that GLIPH can be used independently of knowing epitope specificity to elucidate novel clusters within repertoires it has not been exposed to previously. Even though GLIPH was validated across patients, it is yet unclear whether or not it will be able to cluster TCRs based on their epitope preference in a mixed epitope repertoire with unknown specificities.

## 4 Summary and outlook

In order to evaluate the performance of the sequence based methods we performed a preliminary comparison using data obtained from VDJdb database taking all human *β*TCRs paired with their epitope specificities with a VDJdb confidence score above 1. This dataset was split into training and testing datasets based on epitope similarity, so that there are no shared epitopes between the two. The testing set finally consisted of 830 TCRs with known specificity towards one of 28 epitopes. We assessed each method as binary classifiers, based on their ability to cluster together TCRs with identical specificity, and measured their accuracy in terms of Area Under the Roc curve (AUC) [48]. The AUC is 1 for a perfect prediction, and 0.5 for a random prediction. TCRdist was not evaluated as it is calculated considering paired *α* and *β* TCR chains simultaneously. Immunomap and CDRdist performed comparably, with an AUC of 0.6449 and 0.6502, respectively. However, when we performed an agglomerative (“bottom-up”) hierarchical clustering [49] approach the methods did not reveal any epitope specific clusters. These results are not surprising since both of these methods are based on sequence alignment and scoring techniques on the CDR_*β*_3 region, which is both variable in length and sequence. As mentioned in the introduction, TCRs with very different sequences can bind to the same epitope, and both methods fail at identifying such cases and at forming epitope-specific clusters.

TCRdist contains also information on the CDR1 and CDR2, which come into close contact with the MHC complex. As MHCs also exhibit preference in epitope presentation [50], [51], this provides additional information with respect to methods focused solely on the CDR3 region. Furthermore, TCRdist combines both the alpha and beta chain regions in its analysis, possibly increasing the sensitivity of the method, as both chains are involved in pMHC recognition. On the other hand, this comes at an additional cost, since paired sequencing is still less abundant than bulk sequencing data. Nevertheless, all sequence alignment techniques carry an inherent fault since they can introduce gaps in the sequences at different positions, rather than focusing on structurally conserved regions in the CDRs that mediate epitope recognition.

The short motif search method has shown remarkable power considering that it does not include entire TCR sequences in the comparison. The short motifs considered are expected to convey a notion of conserved stretches of amino acid sequence coming into contact with the epitope. Which is precisely what the alignment methods are struggling to capture. A difficulty arising in this analysis is that choice of motif length is quite arbitrary. Furthermore, both reviewed analysis focus solely on the CDR_*β*_3 region. Even though GLIPH uses scoring matrices to evaluate similarity of the motifs found in CDR3s, when evaluated on a mixture of eight CDR3 specificities it is not able to cluster all TCRs. Out of the TCRs that were clustered GLIPH is able to group them according to their epitope cluster with 94% accuracy. This remarkable results possibly stems from the fact that epitopes can be evolutionary related, and therefore the short motifs specific to them can in theory reflect this evolutionary similarity. Furthermore, the GLIPH algorithm takes in simultaneously both local motif and global similarity of TCRs capturing more complex characteristics of TCRs. GLIPH is yet to be evaluated on it’s predicting power on clustering all the TCRs in the mixture of TCRs with known specificites.

Currently no single tool exists for unequivocal classification of TCR receptor specificity. This is due to two major biological features of the data. Firstly, TCRs are cross-reactive and able to bind multiple antigens with varying affinities. Furthermore, TCR binding is not sufficient to elicit T cell activation. A complex interplay between binding affinity and stability, co-stimulatory signals, and TCR abundancy regulates T cell activation [52]. This underlines the complexity of a T cell antigen response, meaning that clustering to predict epitope specificity might not necessarily show the true state of epitope reactivity. This potentially hampers the intended use of these methods in disease outcome predictions.

Secondly, TCR data, especially CDR3 regions, carry innate redundancy as the termini of CDR3s across individuals share high sequence similarity, that leaves a short stretch of CDR3 sequence responsible for such a high variability in epitope binding. This similarity comes from V and J genes shared across TCRs and the nature of V(D) J recombination which introduces most sequence variability in the junctions between the individual genes. Upon training a classification method or constructing a similarity metric with the aim of elucidating epitope specificity, much of the dataset will share high similarity with the testing set. Therefore the performance of these methods might plummet dramatically in real-life applications. One possible way to overcome this is by obtaining larger quantities of data than available at present. Higher throughput of technologies which pair TCRs with epitopes, such as multimer technologies, might provide the data necessary to train the more complex machine learning algorithms such as neural networks, to achieve better performance.

Additionally, TCR epitope recognition in reality occurs in three-dimensional space, therefore understanding the complex TCR-pMHC interaction from primary sequence alone is challenging. The importance of including 3D structural information in models for TCR target prediction has already been recognised [53]. Therefore including TCR structural information into clustering approaches might greatly improve prediction of epitope specificities.

Overall, the rise of availability of bulk and paired *αβ* TCR sequencing data offers the opportunity to improve the methods to cluster TCRs and predict their epitope specificities. As TCR data becomes more abundant, the need for higher computing power will rise too. Currently, methods are usually limited to assessing samples of up to 100,000 unique TCR sequences at a time, with subsampling techniques readily employed to increase the analysis speed. When we reach the aspired goal for the amount of TCR epitope annotated data, the machines currently available to most researchers will not carry sufficient computational power to perform such tasks. However, technological advances will ensue, which will allow even more computing power to be readily available to a wide population of scientists and empower researchers for even larger scale data analysis.

## Declaration of Competing Interest

The authors declare that they have no known competing financial interests or personal relationships that could have appeared to influence the work reported in this paper.

## CRediT authorship contribution statement

**Milena Vujovic:** Conceptualization, Writing - original draft, Writing - review & editing, Visualization. **Kristine Fredlund Degn:** Software, Formal analysis. **Frederikke Isa Marin:** Writing - review & editing. **Anna-Lisa Schaap-Johansen:** Visualization. **Benny Chain:** Writing - review & editing. **Thomas Lars Andresen:** Writing - review & editing, Supervision, Funding acquisition. **Joseph Kaplinsky:** Conceptualization, Writing - original draft. **Paolo Marcatili:** Conceptualization, Writing - original draft, Supervision, Project administration.

## References

[1] Mamedov I.Z., Britanova O.V., Zvyagin I.V., Turchaninova M.A., Bolotin D.A., Putintseva E.V., Lebedev Y.B., Chudakov D.M. (2013). Preparing unbiased T-cell receptor and antibody cDNA libraries for the deep next generation sequencing profiling. Front Immunol.

[2] Leinonen R., Sugawara H., Shumway M., o. b. o. t. I.N.S.D. Collaboration (2010). The sequence read archive. Nucl Acids Res.

[3] Shugay M., Bagaev D.V., Zvyagin I.V., Vroomans R.M., Crawford J.C., Dolton G., Komech E.A., Sycheva A.L., Koneva A.E., Egorov E.S., Eliseev A.V., VanăDyk E., Dash P., Attaf M., Rius C., Ladell K., McLaren J.E., Matthews K.K., Clemens E., Douek D.C., Luciani F., vanăBaarle D., Kedzierska K., Kesmir C., Thomas P.G., Price D.A., Sewell A.K., Chudakov D.M. (2017). VDJdb: a curated database of T-cell receptor sequences with known antigen specificity. Nucl Acids Res.

[4] Gowthaman R., Pierce B.G. (2019). TCR3d: The T cell receptor structural repertoire database. Bioinformatics.

[5] immuneACCESS Data; 2020. URL: https://clients.adaptivebiotech.com/immuneaccess.

[6] Memarnejadian A., Meilleur C.E., Shaler C.R., Khazaie K., Bennink J.R., Schell T.D., Haeryfar S.M.M. (2017). PD-1 blockade promotes epitope spreading in anticancer CD8 + T cell responses by preventing fratricidal death of subdominant clones to relieve immunodomination. J Immunol.

[7] Gulley J.L., Madan R.A., Pachynski R., Mulders P., Sheikh N.A., Trager J. (2017). Role of antigen spread and distinctive characteristics of immunotherapy in cancer treatment. J Natl Cancer Inst.

[8] Murphy K.K.M., Weaver C. (2016). Janeway’s immunobiology.

[9] Abelin J.G., Keskin D.B., Sarkizova S., Hartigan C.R., Zhang W., Sidney J., Stevens J., Lane W., Zhang G.L., Eisenhaure T.M., Clauser K.R., Hacohen N., Rooney M.S., Carr S.A., Wu C.J. (2017). Mass spectrometry profiling of HLA-associated peptidomes in mono-allelic cells enables more accurate epitope prediction. Immunity.

[10] Trolle T., McMurtrey C.P., Sidney J., Bardet W., Osborn S.C., Kaever T., Sette A., Hildebrand W.H., Nielsen M., Peters B. (2016). The length distribution of class I-restricted T cell epitopes is determined by both peptide supply and MHC allele-specific binding preference. J Immunol.

[11] Laydon D.J., Bangham C.R., Asquith B. (1675). Estimating T-cell repertoire diversity: limitations of classical estimators and a new approach. Philos Trans R Soc B: Biol Sci.

[12] Davis MM, Bjorkman PJ. T-cell antigen receptor genes and T-cell recognition; 1988.https://doi.org/10.1038/334395a0. 10.1038/334395a0 3043226

[13] Jenkins M.K., Chu H.H., McLachlan J.B., Moon J.J. (2010). On the composition of the preimmune repertoire of T cells specific for Peptide-major histocompatibility complex ligands. Annu Rev Immunol.

[14] Sewell AK. Why must T cells be cross-reactive?; 2012.https://doi.org/10.1038/nri3279. 10.1038/nri3279 PMC7097784 22918468

[15] Jensen K.K., Rantos V., Jappe E.C., Olsen T.H., Jespersen M.C., Jurtz V., Jessen L.E., Lanzarotti E., Mahajan S., Peters B., Nielsen M., Marcatili P. (2019). TCRpMHCmodels: structural modelling of TCR-pMHC class I complexes. Scientific Rep..

[16] Leem J., deăOliveira S.H., Krawczyk K., Deane C.M. (2017). STCRDab: the structural T-cell receptor database. Nucl Acids Res.

[17] Lanzarotti E., Marcatili P., Nielsen M. (2018). Identification of the cognate peptide-MHC target of T cell receptors using molecular modeling and force field scoring. Mol Immunol.

[18] Pierce B.G., Weng Z. (2013). A flexible docking approach for prediction of T cell receptor-peptide-MHC complexes. Protein Sci.

[19] Liu I.H., Lo Y.S., Yang J.M. (2013). Genome-wide structural modelling of TCR-pMHC interactions. BMC Genomics.

[20] Hoffmann T., Marion A., Antes I. (2017). DynaDom: structure-based prediction of T cell receptor inter-domain and T cell receptor-peptide-MHC (class I) association angles. BMC Struct Biol.

[21] Sedgwick J.D., Holt P.G. (1986). The ELISA-plaque assay for the detection and enumeration of antibody-secreting cells. An overview. J Immunol Methods.

[22] Anthony D.D., Lehmann P.V. (2003). T-cell epitope mapping using the ELISPOT approach. Methods.

[23] Altman JD, Moss PAH, Goulder PJR, Barouch DH, McHeyzer-Williams MG, Bell JI, McMichael AJ, Davis MM. Phenotypic analysis of antigen-specific T lymphocytes. Science 274; 1996: 94–96. J Immunol (Baltimore, Md.: 1950) 187(1); 2011: 7–9. URL:https://pubmed.ncbi.nlm.nih.gov/21690331. 21690331

[24] Andersen R.S., Kvistborg P., Mørch Frøsig T., Pedersen N.W., Lyngaa R., Bakker A.H., Shu C.J., Straten P.T., Schumacher T.N., Hadrup S.R. (2012). Parallel detection of antigen-specific t cell responses by combinatorial encoding of MHC multimers. Nat Protoc.

[25] Hadrup S.R., Bakker A.H., Shu C.J., Andersen R.S., van Veluw J., Hombrink P., Castermans E., thor Straten P., Blank C., Haanen J.B., Heemskerk M.H., Schumacher T.N. (2009). Parallel detection of antigen-specific T-cell responses by multidimensional encoding of MHC multimers. Nat Methods.

[26] Klausen M.S., Anderson M.V., Jespersen M.C., Nielsen M., Marcatili P., LYRA (2015). a webserver for lymphocyte receptor structural modeling. Nucl Acids Res.

[27] Abbas A.K., Lichtman A.H., Pillai S., Baker D.L.M.i., Baker A. (2017). Cellular and molecular immunology.

[28] Schrödinger LLC. The PyMOL Molecular Graphics System, Version1.8; 2015.

[29] TCR-Antigen Map – Adaptive Biotechnologies; 2020. URL: https://www.adaptivebiotech.com/partnerships/antigen-map/.

[30] Emerson R.O., DeWitt W.S., Vignali M., Gravley J., Hu J.K., Osborne E.J., Desmarais C., Klinger M., Carlson C.S., Hansen J.A., Rieder M., Robins H.S. (2017). Immunosequencing identifies signatures of cytomegalovirus exposure history and HLA-mediated effects on the T cell repertoire. Nat Genet.

[31] Henikoff S., Henikoff J.G. (1992). Amino acid substitution matrices from protein blocks. Proc Natl Acad Sci USA.

[32] Dayhoff MO, Dayhoff MO, Schwartz RM. Chapter 22: a model of evolutionary change in proteins. In Atlas of protein sequence and structure. URL: http://citeseerx.ist.psu.edu/viewdoc/summary?doi=10.1.1.145.4315.

[33] Needleman S.B., Wunsch C.D. (1970). A general method applicable to the search for similarities in the amino acid sequence of two proteins. J Mol Biol.

[34] Smith T.F., Waterman M.S. (1981). Identification of common molecular subsequences. J Mol Biol.

[35] Gotoh O. (1982). An improved algorithm for matching biological sequences. J Mol Biol.

[36] Sidhom J.W., Bessell C.A., Havel J.J., Kosmides A., Chan T.A., Schneck J.P. (2018). ImmunoMap: a bioinformatics tool for T-cell repertoire analysis. Cancer Immunol Res.

[37] Dash P., Fiore-Gartland A.J., Hertz T., Wang G.C., Sharma S., Souquette A., Crawford J.C., Clemens E.B., Nguyen T.H.O., Kedzierska K., La Gruta N.L., Bradley P., Thomas P.G. (2017). Quantifiable predictive features define epitope-specific T cell receptor repertoires. Nature.

[38] Thakkar N., Bailey-Kellogg C. (2019). Balancing sensitivity and specificity in distinguishing TCR groups by CDR sequence similarity. BMC Bioinf.

[39] Zvyagin I.V., Pogorelyy M.V., Ivanova M.E., Komech E.A., Shugay M., Bolotin D.A., Shelenkov A.A., Kurnosov A.A., Staroverov D.B., Chudakov D.M., Lebedev Y.B., Mamedov I.Z. (2014). Distinctive properties of identical twins’ TCR repertoires revealed by high-throughput sequencing. Proc Natl Acad Sci USA.

[40] Glanville J., Huang H., Nau A., Hatton O., Wagar L.E., Rubelt F., Ji X., Han A., Krams S.M., Pettus C., Haas N., Arlehamn C.S., Sette A., Boyd S.D., Scriba T.J., Martinez O.M., Davis M.M. (2017). Identifying specificity groups in the T cell receptor repertoire. Nature.

[41] Tickotsky N., Sagiv T., Prilusky J., Shifrut E., Friedman N. (2017). McPAS-TCR: a manually curated catalogue of pathology-associated T cell receptor sequences. Bioinformatics (Oxford, England).

[42] Clackson T., Wells J.A. (1995). A hot spot of binding energy in a hormone-receptor interface. Science.

[43] Ovchinnikov S., Kamisetty H., Baker D. (2014). Robust and accurate prediction of residue-residue interactions across protein interfaces using evolutionary information. eLife.

[44] Marks DS, Hopf TA, Sander C. Protein structure prediction from sequence variation; 2012.https://doi.org/10.1038/nbt.2419. 10.1038/nbt.2419 PMC4319528 23138306

[45] Thomas N., Best K., Cinelli M., Reich-Zeliger S., Gal H., Shifrut E., Madi A., Friedman N., Shawe-Taylor J., Chain B. (2014). Tracking global changes induced in the CD4 T-cell receptor repertoire by immunization with a complex antigen using short stretches of CDR3 protein sequence. Bioinformatics (Oxford, England).

[46] Atchley W.R., Fitch W.M., Fernandes A.D., Drüke T. (1997). A natural classification of the basic helix-loop-helix class of transcription factors. Proc Natl Acad Sci USA.

[47] Cortes C., Vapnik V. (1995). Support-vector networks. Mach Learn.

[48] Swets J.A. (1988). Measuring the accuracy of diagnostic systems. Science.

[49] Nielsen F. (2016). Hierarchical clustering. Undergraduate topics in computer science.

[50] Jurtz V., Paul S., Andreatta M., Marcatili P., Peters B., Nielsen M. (2017). NetMHCpan-4.0: improved peptide–MHC class I interaction predictions integrating eluted ligand and peptide binding affinity data. J Immunol.

[51] Jurtz V.I., Jessen L.E., Bentzen A.K., Jespersen M.C., Mahajan S., Vita R., Jensen K.K., Marcatili P., Hadrup S.R., Peters B., Nielsen M. (2018). NetTCR: sequence-based prediction of TCR binding to peptide-MHC complexes using convolutional neural networks. bioRxiv.

[52] Gálvez J., Gálvez J.J., García-Peñarrubia P. (2019). Is TCR/pMHC affinity a good estimate of the T-cell response? An answer based on predictions from 12 phenotypic models. Front Immunol.

[53] Lanzarotti E, Marcatili P, Nielsen M. T-cell receptor cognate target prediction based on paired *α* and *β* chain sequence and structural CDR loop similarities. Front Immunol 10; 2019: 2080.https://doi.org/10.3389/fimmu.2019.02080. 10.3389/fimmu.2019.02080 PMC6724566 31555288

